# Diva/BclB regulates differentiation by inhibiting NDPKB/Nm23H2-mediated neuronal differentiation in PC-12 cells

**DOI:** 10.1186/1471-2202-13-123

**Published:** 2012-10-11

**Authors:** Jasmin Qian Ru Lim, Jia Lu, Bei Ping He

**Affiliations:** 1 Department of Anatomy, Yong Loo Lin School of Medicine, National University of Singapore, 117597, Singapore; 2 Defence Medical and Environmental Research Institute, DSO National Laboratories (DMERI@DSO), 27 Medical Drive, Singapore, 117510, Singapore

**Keywords:** Diva/BclB, NDPKB/Nm23H2, Differentiation, Neuritogenesis, Proliferation

## Abstract

**Background:**

Diva (*d*eath *i*nducer binding to *v*Bcl-2 and *A*paf-1)/BclB is a Bcl-2 family member, which is known for its function in apoptosis. Diva/BclB has been shown to interact with NDPKB/Nm23H2, which is involved in cellular differentiation. Thus far, there has been no direct evidence of Diva/BclB having a role in differentiation. In the present study, we investigated the expression of Diva/BclB and NDPKB/Nm23H2 during differentiation in PC-12 cell line.

**Results:**

Our results show that after differentiation, Diva/BclB expression was decreased and reciprocally, NDPKB/Nm23H2 expression was increased and it translocated into the nucleus. Overexpression of NDPKB/Nm23H2 promoted PC-12 neuronal differentiation by increasing neurite outgrowth and arresting cell cycle progression. There was a concurrent downregulation of Diva/Boo when NDPKB/Nm23H2 was overexpressed, which mirrors the effect of NGF on PC-12 cell differentiation. Overexpression of Diva/BclB did not change the expression level of NDPKB/Nm23H2, but inhibited its nuclear localization. Cells that overexpressed Diva/BclB presented a decreased percentage of differentiated cells and average neurite length was shortened. This was due to an increase in the formation of Diva/BclB and NDPKB/Nm23H2 complexes as well as Diva/BclB and β-tubulin complexes. Concomitantly, there was a decrease in formation of NDPKB/Nm23H2 and β-tubulin complexes. Overexpression of Diva/BclB also resulted in a higher percentage of S-phase cells.

**Conclusion:**

Our results showed a novel role for Diva/BclB in neuronal differentiation. Its downregulation during neuronal differentiation may be necessary to allow NDPKB/Nm23H2 and β-tubulin interaction that promotes NDPKB/Nm23H2 mediated differentiation.

## Background

The PC-12 cell line has been used extensively as a model of neuronal differentiation, as the cells halt proliferation, have outgrowth of neurites and acquire properties of sympathetic neurons after exposure to nerve growth factor (NGF) [1]. The differentiation response starts with the stimulation of receptor tyrosine kinase (TrkA) which activates the intracellular signal transduction cascades, including those mediated by Raf-MAPK kinase-ERK and other pathways regulated by Rho family of small GTPases [2]. During the last few years, there have also been reports of negative regulators of neuronal differentiation in PC-12 cells, such as Sprouty (Spry) family of proteins and Idh3α [3,4].

Diva/BclB is a member of the Bcl-2 family of proteins. A human ortholog has also been identified, and designated as BclB or Bcl2l10. It is also known as Boo (Bcl-2 homologue of ovary). Diva/BclB contains BH1, BH2, BH3, and BH4 regions and a carboxyl-terminal hydrophobic domain. It is distributed in the brain, liver, and heart of E15 embryonic mice. Interestingly, in adult mice the expression of Diva/BclB decreases dramatically and becomes restricted to the granulosa cells of the ovary and the seminiferous tubules of the testis [5]. Diva/BclB labeling in seminiferous tubules is consistent with a stage-specific expression during spermatogenesis. Thus far, most reports on Diva/BclB have reported on its effect on apoptosis. Diva/BclB has been reported to form a ternary complex with apoptosis-activating factor-1 (Apaf-1) and caspase-9, to induce BH-3 independent cell death [5]. However, Boo was reported to inhibit apoptosis through homodimerization or heterodimerization with some pro-apoptotic (Bak and Bid) and anti-apoptotic (Bcl-2 and Bcl-xl) Bcl-2 family members [6]. More recently, Diva/BclB was shown to mediate cell death through interaction with a nucleoside diphosphate kinase (NDPK) isoform, NM23-H2/NDPK B [7].

Nm23/NDPK are house keeping enzymes needed for transfer of the terminal phosphate of a nucleoside triphosphate to a nucleoside diphosphate. Out of the ten known human Nm23 family genes, NDPKA/Nm23H1 and NDPKB/Nm23H2 are the most abundant and ubiquitous isoforms, and are located both in the nuclei and cytoplasm [8]. Apart from its kinase activity, Nm23/NDPK has been suggested to be involved in a variety of cellular functions including differentiation, proliferation, gene regulation and apoptosis [9-11]. Overexpression of Nm23-M1 protein induced neurite outgrowth and increased the expression of neurofilament and microtubule proteins, whereas downregulation of Nm23/NDPK enhanced cell proliferation and inhibited neuronal differentiation [12].

Thus far, the physiological role of Diva/BclB has remained elusive due to a paucity of information. Even its function in apoptosis is highly controversial with many opposing reports. Recently, there has been evidence of Diva/BclB having a direct role in development. Knockdown of the zebrafish ortholog (NRZ) resulted in disorganized somites, increased levels of Snail-1, and disorganisation of the microtubule network and F-actin depolymerisation in the yolk sac [13,14]. Diva/BclB has a decreased expression pattern during development, and is known to interact with NDPKB/Nm23H2 which is involved in cellular differentiation. These findings prompted us to investigate if Diva/BclB also has a role in cellular differentiation. Using overexpression studies, we demonstrated that Diva/BclB negatively regulates PC-12 neuronal differentiation, by affecting neurite outgrowth and cell proliferation. Furthermore, we report that Diva/BclB mediates these processes through its interaction and regulation of subcellular localization of NDPK B/Nm23H2.

## Results

### Up-regulation of NDPKB and down-regulation of Diva during neuronal differentiation

**Figure 1:**
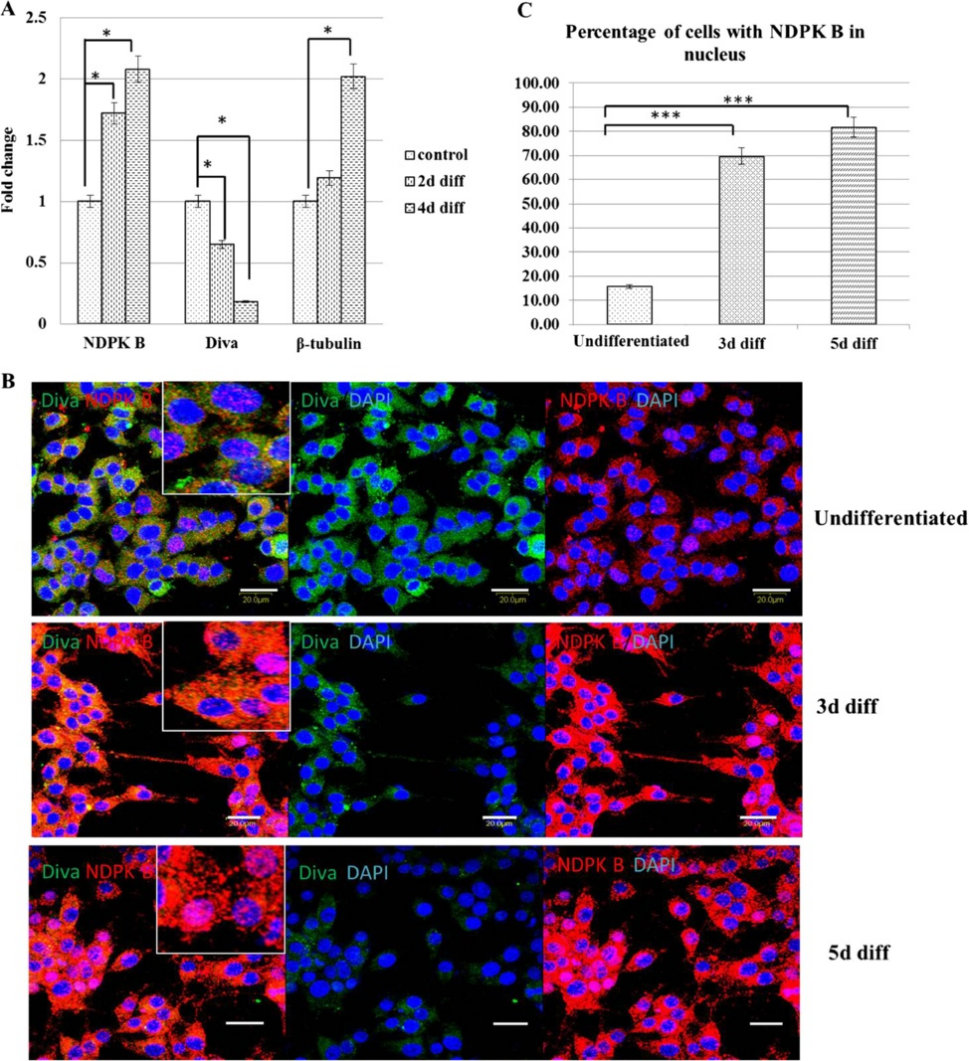
**Reciprocal expression of Diva/BclB and NDPKB/Nm23H2 proteins in differentiated PC-12 cells**. (**A**) Real time-PCR showed decreased expression of Diva/BclB mRNA and upregulation of NDPKB/Nm23H2 and β-tubulin expressions. The data was normalized using GAPDH expression. (**B**) Western blotting show protein levels follow the same trend after differentiation. (**C**) Immunocytochemistry staining with Diva/BclB (green) and NDPKB/Nm23H2 (red) antibodies show colocalization of both proteins in the cytoplasm. (**D**) Nuclear staining of NDPK B was observed in differentiated cells, and the percentage of cells with NDPKB/Nm23H2 in the nucleus was quantified after counting an average of 300 cells. Bar=40μm; *p<0.05, ***p<0.001.

To address whether NDPKB/Nm23H2 and Diva/BclB may play physiological roles in neuronal differentiation, we examined the expression of these proteins during PC-12 neuronal differentiation. PC-12 cells showed a steady and significant increase in NDPKB/Nm23H2 and β-tubulin mRNA levels, with a concurrent decrease in Diva/BclB mRNA levels during the course of NGF-induced differentiation (Figure 1A). Detection of protein expressions using immunocytochemistry also showed the up-regulation of NDPKB/Nm23H2 and down-regulation of Diva/BclB (Figure 1B).

In the cells not treated with NGF, the distribution of Diva/BclB was detected to be in the cytoplasm, and NDPKB/Nm23H2 was mostly in the cytoplasm with some cells staining positive for the nucleus as well. By 3 days after NGF-induced differentiation, the cells expressed a lesser amount of Diva/BclB and this low expression was maintained at 5 days after differentiation. In contrast, differentiated cells stained more intensely for NDPKB/Nm23H2 after 3 days of differentiation and remain elevated even at 5 days, and a greater percentage of cells showed staining for the nucleus as compared to the control (Figure 1B). Statistical analysis showed the percentage of cells with NDPKB/Nm23H2 B in the nucleus was significantly elevated after the cells differentiate (Figure 1C).

### NDPKB/Nm23H2 protein promotes outgrowth of neurites

**Figure 2:**
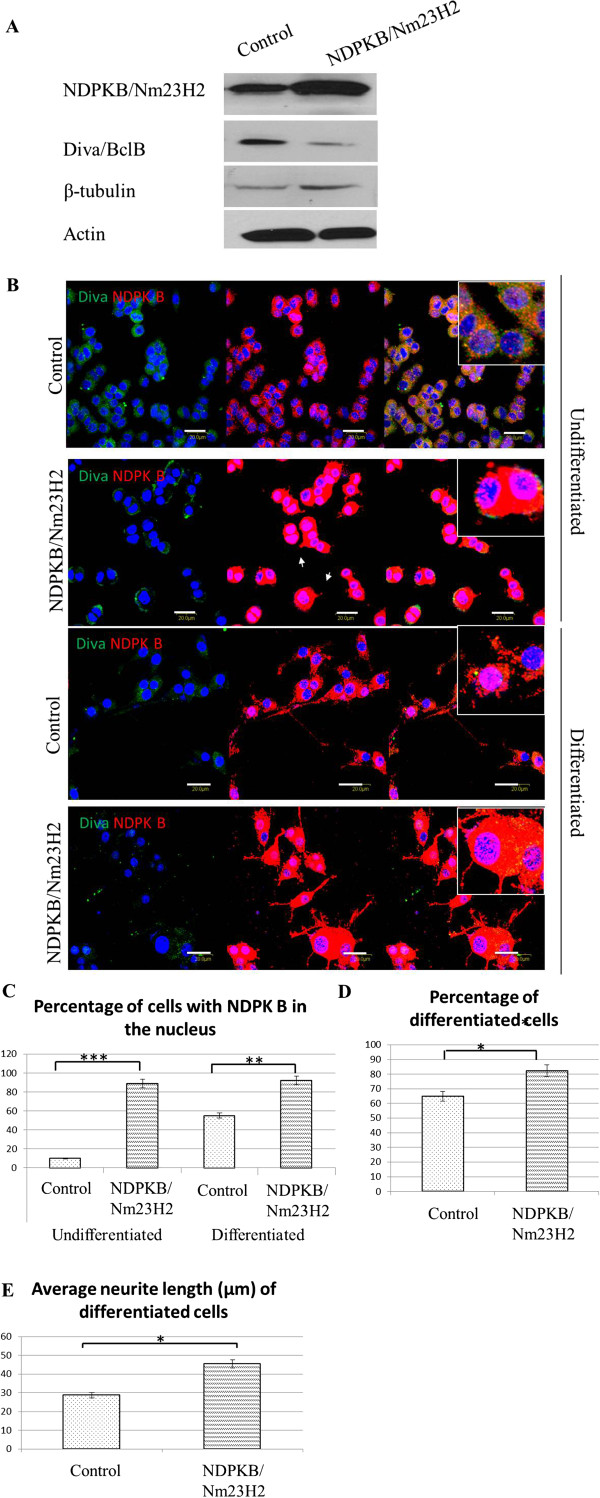
**Overexpression of NDPKB/Nm23H2 increases cell differentiation and neurite length**. The PC-12 cells were harvested or fixed 24hrs post-transfection. (**A**) Immunoblotting showed that overexpression of NDPKB/Nm23H2 suppresses expression of Diva/BclB. (**B**) Immunocytochemistry staining with Diva/BclB (green) and NDPKB/Nm23H2 (red) antibodies show colocalization of both proteins in the cytoplasm. (**C**) The percentages of cells with NDPKB/Nm23H2 in the nucleus were greatly elevated after the protein was overexpressed. Both the percentage of differentiated cells (**D**) and average neurite length (**E**) of the differentiated cells were increased in the NDPKB/Nm23H2 overexpressing cells. An average of 150 cells per group was counted for graphical analyses, and the experiments were performed in triplicate (Bar=40μm, *p<0.05, **p<0.01, ***p<0.001).

In order to investigate if the increased expression NDPKB/Nm23H2 were directly involved in the process of differentiation, we overexpressed NDPKB/Nm23H2 in the PC-12 cell line. For this purpose, PC-12 cells were transfected with 2μg of control and NDPKB/Nm23H2 plasmids. Quantification of protein levels (Figure 2A) showed that overexpression of NDPKB/Nm23H2 resulted in a significant increase in NDPKB/Nm23H2 levels, upregulation in β-tubulin levels, and downregulation in Diva/BclB expression. This is similar to the expression profile of these proteins during NGF-induced differentiation.

The ectopic NDPKB/Nm23H2 protein was expressed in both nucleus and cytoplasm of the transfected cells (Figure 2B). Even without NGF treatment, the NDPKB/Nm23H2 overexpressing cells also had little branches appearing, suggesting that NDPKB/Nm23H2 might help to initiate neuronal differentiation. When exposed to NGF, the cells overexpressing NDPKB/Nm23H2 also had a more branched morphology, with greater outgrowth of neurites (Figure 2B). Ectopic NDPKB/Nm23H2 was expressed in both nucleus and cytoplasm of the transfected cells, therefore leading to significant increases in the percentages of undifferentiated and differentiated cells with NDPKB/Nm23H2 in their nucleus (Figure 2C). Cells that overexpressed NDPKB/Nm23H2 had 82.4±2.81% differentiated cells, which was significantly more than the 64.86±3.39% in the control (Figure 2D). They also had longer neurites, measuring 35.42.6 ± 1.54 μm as compared to 28.74 ± 1.55 μm in control cells (Figure 2E). Therefore, this shows that upregulation of NDPKB/Nm23H2 during differentiation facilitates neurite outgrowth.

### Diva/BclB inhibits neurite outgrowth and prevents the translocation of NDPKB/Nm23H2 into the nucleus during differentiation

**Figure 3:**
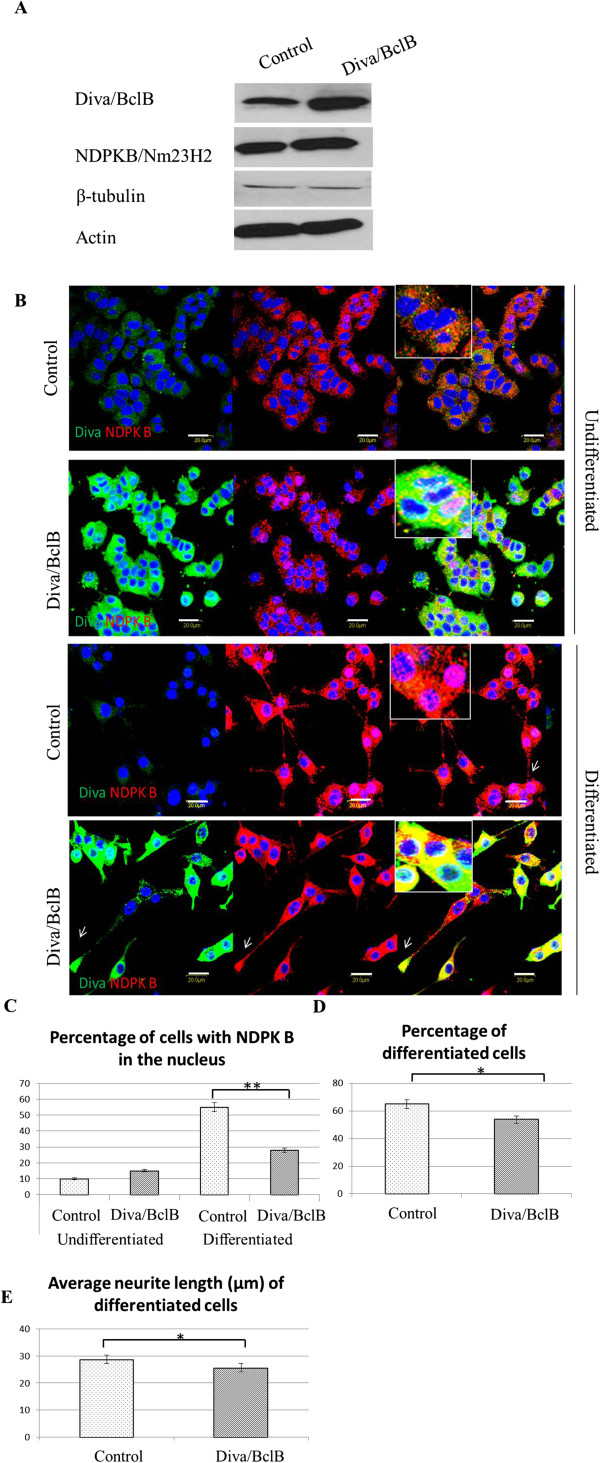
**Overexpression of Diva/BclB decreases cell differentiation and neurite length**. After transfection with either the empty vector (control) or Diva/BclB overexpressing plasmid(Diva/BclB), immunoblotting (**A**) both showed an increase in Diva/BclB expression without any change to NDPKB/Nm23H2 expression. (**B**) Overexpression of Diva (green) resulted in stronger immunostaining in the cytoplasm. There was no change in NDPKB/Nm23H2 (red) expression. In the Diva/BclB cells, Diva/BclB expression remained strong even after 24 hrs of differentiation. There was strong colocalization of both proteins in the cytoplasm and at the neurite. (**C**) Overexpression of Diva/BclB prevents translocation of NDPKB/Nm23H2 into the nucleus after differentiation. It also decreases the percentage of differentiated cells (**D**) and their average neurite length (**E**). An average of 150 cells per group was counted for graphical analyses, and the experiments were performed in triplicate (Bar =40μm, *p<0.05, **p<0.01).

Since NGF-induced differentiation of PC-12 cells as well as NDPKB/Nm23H2 overexpression resulted in downregulation of Diva/BclB expression, we next investigated if Diva/BclB might be a negative regulator of neuronal differentiation. For this purpose, PC-12 cells were transfected with control or Diva/BclB plasmid. Overexpression of Diva did not affect the protein expression level of β-tubulin and NDPKB/Nm23H2 (Figure 3A). In the undifferentiated cells, overexpression of Diva/BclB resulted in a slight but non-significant increase in percentage of cells with NDPKB/Nm23H2 in the nucleus, so the majority of the protein was still expressed in the cytoplasm (Figure 3B). However, in the differentiated cells, ectopic expression of Diva/BclB affected NDPKB/Nm23H2 localization and cellular morphology. Cells with strong expression of Diva/BclB had shorter neurites with strong co-localization of Diva/BclB and NDPKB/Nm23H2 at the neurite terminal (white arrow), whereas cells with downregulated Diva/BclB expression had long neurites. Diva/BclB overexpressing cells had 53.76±3.75% differentiated cells, which was significantly lesser than the 64.86±3.39% in the control (p<0.05) (Figure 3D). Diva/BclB overexpressing cells also had shorter neurites, measuring 25.6 ± 0.66μm as compared to 28.74 ± 1.55 μm in control cells (p<0.05) (Figure 3E).

While the control cells that were differentiated had translocation of NDPKB/Nm23H2 to the nucleus, cells that were overexpressing Diva/BclB had most of their nucleus unstained with the NDPKB/Nm23H2 antibody despite differentiation (Figure 3B). Analysis showed that the percentage of differentiated cells with NDPKB/Nm23H2 in their nucleus was lesser in Diva-overexpressing cells (Figure 3C), indicating that Diva might be sequestering NDPKB/Nm23H2 in the cytoplasm.

### Diva/BclB inhibits neurite outgrowth by preventing the association of NDPKB/Nm23H2 and β-tubulin

As it is known that β-tubulin is a component of microtubules, and that neuritogenesis requires changes to the cytoskeletal network, we studied the interactions of Diva/BclB, NDPKB/Nm23H2 and β-tubulin to further understand the mechanism through which Diva/BclB inhibited neurite outgrowth. Using Duolink kit, which is a proximity ligation assay kit, we examined the subcellular locations and amounts of Diva/BclB and NDPKB/Nm23H2, NDPKB/Nm23H2 and β-tubulin as well as Diva/BclB and β-tubulin complexes.

**Figure 4:**
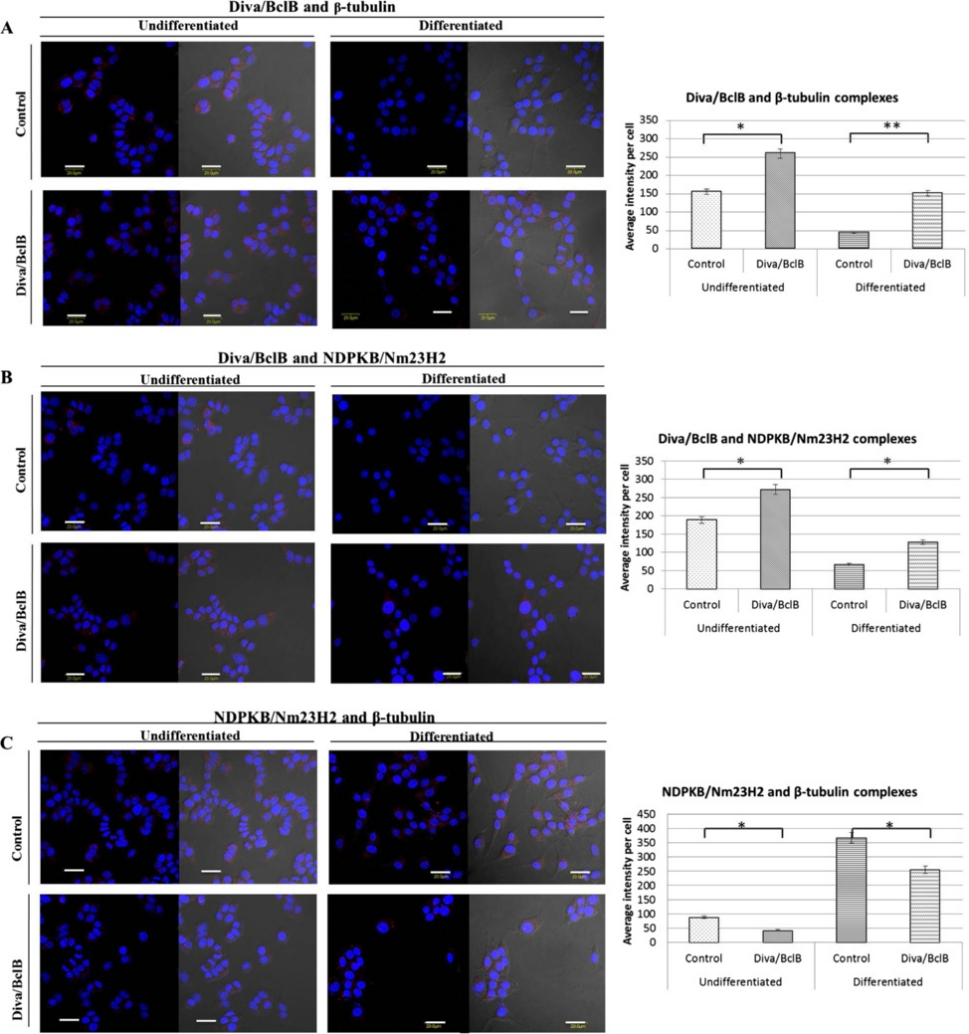
**Diva/BclB prevents NDPKB/Nm23H2 and β-tubulin complex formation**. After transfection, the cells were grown for a day under normal growth medium or differentiating medium for 24 hrs. The interaction between the proteins were analysed using the Duolink assay. Regardless of the differentiation status, there were more Diva/BclB and β-tubulin (**A**) and Diva/BclB and NDPKB/Nm23H2 (**B**) complexes, and fewer NDPKB/Nm23H2 and β tubulin (**C**) complexes formed in Diva-overexpressing cells. (Bar= 20; *p<0.05, **p<0.01).

Overexpression of Diva/BclB led to increased amounts of Diva/BclB and NDPKB/Nm23H2, as well as Diva/BclB and β-tubulin complexes in undifferentiated cells. This was maintained at a relatively high number even after differentiation (Figure 4A & B). In contrast, overexpression of Diva/BclB resulted in fewer NDPKB/Nm23H2 and β-tubulin complexes in both undifferentiated and differentiated cells (Figure 4C). Hence, the interaction between Diva/BclB and NDPKB/Nm23H2 would retain NDPKB/Nm23H2 in the cytosol and prevent interaction of NDPKB/Nm23H2 and β-tubulin, which then results in decreased neurite outgrowth.

### Diva/BclB increases and NDPKB/Nm23H2 decreases cell proliferation

**Figure 5:**
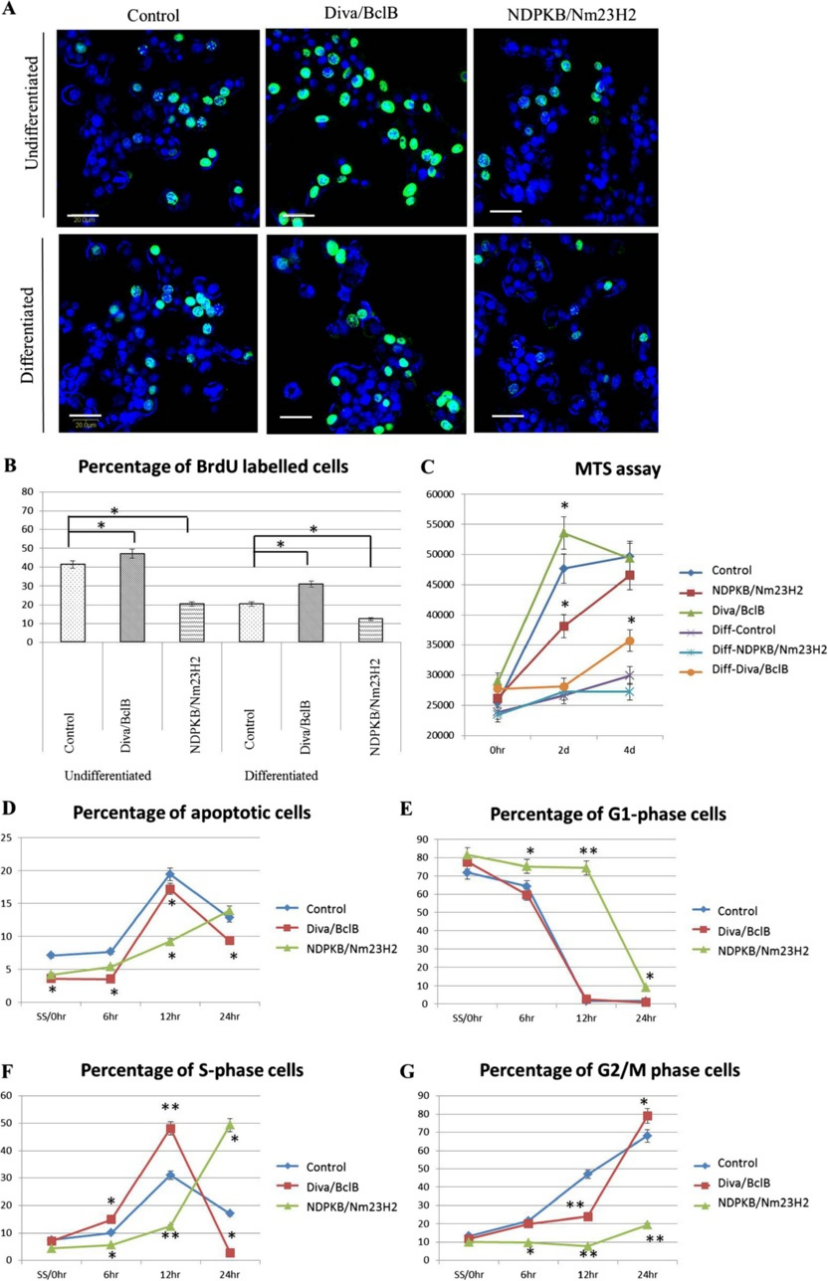
**Effect of Diva/BclB and NDPKB/Nm23H2 on the cell cycle and cellular proliferation of cells.** (**A**) The number of cells in S phase are visualised by BrdU staining (green) in the nucleus. Diva/BclB had the highest proportion of stained nuclei, followed by control and NDPKB/Nm23H2 had the lowest proportion. (**B**) An average of 500 cells per group was counted, and quantitative analysis of the data showed that overexpression of NDPKB/Nm23H2 decreased the percentage of S-phase cells, while Diva/BclB increased cellular proliferation. (**C**) Growth curves of the control and transfected cells were plotted using MTS assay. Diva/BclB cells had a less inhibited growth curve, while NDPKB/Nm23H2 cells had a slower growth curve, when compared to control cells. (**D**-**G**) Flow cytometry data was collected after serum starvation for 24 hours (0hr), and at indicated times after serum addition. Graphical analysis using Summit software showed that overexpression of Diva/BclB resulted in a decrease in apoptotic cells and faster cell cycle progression. In contrast, overexpression of NDPKB/Nm23H2 resulted in slower cell cycle progression. (Bar=20μm, *p<0.05, **p<0.01).

Next, we examined the effects of Diva/BclB and NDPKB/Nm23H2 on cell proliferation as cell cycle exit is one of the hallmarks of neuronal differentiation. To this end, we compared the proliferation rates of the undifferentiated and differentiated transfected cells, by staining them with Brd-U antibodies 24 hours after differentiation (Figure 5A). NDPKB/Nm23H2 overexpressing cells in the control group had a significantly lesser number of cells that were in S phase (Figure 5B). In fact, the number of dividing cells in the undifferentiated NDPKB/Nm23H2 was similar to the differentiated control group, suggesting that increased NDPKB/Nm23H2 expression is responsible for the decreased proliferation during differentiation in normal cells. Overexpression of NDPKB/Nm23H2 was also able to further decrease the number of cycling cells in the differentiated group (Figure 5B). In contrast, Diva/BclB overexpressing cells had a higher percentage of cells nuclei with BrdU staining (Figure 5A & B). Hence, ectopic Diva/BclB expression results in an elevated proliferation rate.

Using MTS assay, growth curves for control, Diva/BclB and NDPKB/Nm23H2 were plotted over a 4 day period (Figure 5C). For the undifferentiated cells, NDPKB/Nm23H2 cells had significantly fewer cells as compared to control, whereas Diva/BclB cells had a peak in cell numbers at 2 days after plating. However, by 4 days all the transfected cells had similar cell numbers, plausibly due to space constraints in the well. The growth curves for all the differentiated transfected cells were attenuated, indicating a slower proliferation rate as compared to the undifferentiated cells. There was a significant increase in cell number of Diva/BclB cells as compared to control cells only 4 days after induction of differentiation. The growth curve for NDPKB/Nm23H2 overexpressing cells was slightly slower than the control cells when under differentiating conditions, but statistical analysis showed no significant difference.

In order to study the effects of both proteins on serum-induced cell cycle re-entry, the transfected cells were serum starved for 24 hours to arrest and synchronize them, before serum was added again to allow cell cycle re-entry. At stipulated time points after re-addition of serum, the cells were stained with propidium iodide and subjected to flow cytometry analysis. Both Diva/BclB and NDPKB/Nm23H2 overexpressing cells had significantly lower percentages of apoptotic cells (Figure 5D). There was a delay in progression from G1 to S-phase for the NDPKB/Nm23H2 cells (Figure 5E) and there was a significant S-phase population only at 24hrs (Figure 5F). The G2/M population for NDPKB/Nm23H2 cells was consistently smaller than the control cells (Figure 5G). In contrast, the Diva/BclB cells had a considerably larger S-phase population than the control at 6 and 12 hrs (Figure 5F). Interestingly, at 12 hours, there was a greater proportion of Diva-overexpressing cells at S-phase, and a lower percentage in the G2/M phase, as compared to the control cells (Figure 5G). Hence, it might signify that Diva has a role in S-phase.

## Discussion

Thus far, reports on Diva/BclB have only covered its role in apoptosis and oocyte maturation [5,15,16]. The results in this study propose a novel function for Diva/BclB, by demonstrating that the protein is involved in the negative regulation of neuronal differentiation, and that it achieves this by inhibiting NDPKB/Nm23H2 function.

In this study, we showed that with NGF-induced differentiation, Diva/BclB expression decreases and NDPKB expression increases. The regulation of these 2 proteins is likely through the sustained ERK signalling by NGF, which results in phosphorylation as well as binding of CREB and AP-1 family members so as to facilitate gene transcription [17,18].

Overexpression of NDPKB/Nm23H2 encouraged PC12 neuronal differentiation by promoting neurite outgrowth and retarding cell proliferation. Interestingly, there was Diva/BclB downregulation in the NDPKB/Nm23H2 overexpressing cells, which is a salient point since decreased expression of Diva/BclB was also observed during NGF-induced differentiation. Thus, this supported that Diva/BclB was a negative modulator of neuronal differentiation that had to be downregulated and suggested that the induced downregulation of Diva/BclB by NDPKB/Nm23H2 could be a positive feedback mechanism for NGF-induced neuronal differentiation.

Overexpression of Diva suppressed NDPKB/Nm23H2 function by retaining NDPKB/Nm23H2 in the cytoplasm, and attenuated the number of NDPKB/Nm23H2 and β-tubulin complexes. In turn, there was an increase in S-phase cells and shorter neurites were observed after NGF-induced neuronal differentiation.

The regulatory molecules that control the rate of neurite growth and the signals that determine when and where the axons and dendrites have to grow are still largely unknown. Overexpression of negative regulators of neuronal differentiation have been shown to block neurite outgrowth and branching by inhibiting the Erk/MAPK and Rac1 but not Akt signaling pathway in response to NGF [3,19]. In our study, Diva/BclB inhibited neurite outgrowth as more Diva/BclB and NDPKB/Nm23H2 as well as Diva/BclB and β-tubulin complexes were formed and the amount of NDPKB/Nm23H2 and β-tubulin complexes decreased significantly. Previous reports have reported the co-immunoprecipitation of NDPK/Nm23 with β-tubulin, and the number of complexes increases during the cellular differentiation process [12,20]. Tubulin phosphorylation by NDPKB/Nm23H2 would allow the microtubules to attach more efficiently to the MAPs and increase microtubule stability, thereby facilitating neurite outgrowth [21-24]. Therefore, this study suggests that the physiological role of Diva/BclB in undifferentiated cells might be to inhibit the association between NDPKB/Nm23H2 and β-tubulin. Expression of Diva/BclB is downregulated during differentiation to allow more NDPKB/Nm23H2 and β-tubulin complexes to form, thereby encouraging neurite outgrowth. This is interesting since Diva/BclB has been shown to interact with Hungtington-interacting protein 1-related (H1P1R) protein and microtubule binding protein, translationally controlled tumor-associated protein (TCTP) and thereby postulated to regulate the cytoskeleton [14,25]. Our study has plugged the gap to show regulation of the cytoskeleton by Diva/BclB and its consequential effect on neurite outgrowth.

In differentiated cells, the overexpression of Diva/BclB in PC-12 cells resulted in intense immunostaining in the cytoplasm, indicating the colocalisation of the 2 proteins, and significantly decreased the number of cells with NDPKB/Nm23H2 in their nucleus. This suggests that overexpression of Diva/BclB was able to sequester NDPKB/Nm23H2 in the cytoplasm and prevent its translocation into the cytoplasm after receiving the signal for differentiation. This is interesting because overexpression of NDPKB/Nm23H2 results in nuclear expression and decreased cellular proliferation. In contrast, the effect of overexpressing Diva/BclB is a higher proliferation rate and decreased nuclear expression of NDPKB/Nm23H2. Hence, this suggests that Diva/BclB is able to regulate proliferation by controlling the nuclear translocation of NDPKB/Nm23H2. NDPKs have been reported to translocate to the nucleus, although there is controversy whether NDPKA/Nm23H1 and NDPKB/Nm23H2 visit the nucleus together or separately [22,26]. Also, reasons for NDPKB/Nm23H2 movement into the nucleus are also unclear, though the translocation has been observed to occur at certain cell phase cycles, thereby suggesting NDPK/Nm23 are required in the nucleus for gene regulation or to supply dNTPs for replication [27]. NDPKB/Nm23H2 has been identified as human transcription factor PuF, a sequence-specific DNA-binding protein with affinity for a nuclease-hypersensitive element (NHE) of the c-MYC gene promoter [28,29]. NDPKB/Nm23H2 has also been demonstrated to be capable of DNA binding, DNA cleavage, as well as transcriptional activity that is independent of phosphoryl transfer and NDPK activity [30]. Thus, we report a novel observation of the translocation of NDPKB/Nm23H2 into the nucleus after differentiation, which causes inhibition of proliferation and retarded cell cycle progression, although the exact mechanisms are yet to be further elucidated.

## Conclusion

In conclusion, our results present a model in which downregulation of Diva/BclB during differentiation is necessary to allow increased formation of NDPKB/Nm23H2 and β-tubulin complexes for neurite outgrowth, and for NDPKB/Nm23H2 to enter the nucleus to inhibit proliferation. Hence this clarifies the developmental observation for the increased NDPKB/Nm23H2 expression seen during the onset of organogenesis in the brain [31], as well as explains the attenuated Diva/BclB expression in the adult brain [5]. Development of the nervous system is a complex process, and the correct expression of Diva and NDPKB/Nm23H2 proteins are important in ensuring regulation of diverse cellular processes including proliferation, differentiation and synaptogenesis.

## Methods

### Cell culture

The PC12 cell line is established from rat adrenal medullary pheochromocytoma has been used as a model for neuronal differentiation. PC12 cells were routinely maintained at 37°C and 5% CO_2_ in DMEM (Sigma Aldrich, USA) supplemented with 10% heat-inactivated fetal bovine serum (FBS) (Hyclone, Germany). To differentiate the cells, 50ng/ml of NGF (Invitrogen, USA) was added to DMEM with 1% FBS (differentiating medium). The differentiating medium was then either added to 1x10^6^ cells in a flask (for real-time PCR and immunoprecipitation studies), or 5x10^5^ cells in a well (for immunocytochemistry studies). The media was replaced with fresh differentiating medium every other day.

### Real-time polymerase chain reaction (RT-PCR)

After differentiation, total RNA was isolated from the cells in each well of a 6-well plate using the RNeasy Mini kit according to the manufacturer’s protocol (Qiagen). The qRT-PCR was performed using a LightCycler instrument (Roche, Germany) and QuantiTect SYBR Green (Qiagen) according to manufacturer’s instructions. The sequences for the primers used are as follows: GAPDH-F (5^1^-AGTCTACTGGCGTCTTCACCA-3^1^), GAPDH-R (5^1^-AGTTGTCATGGATGACCTTGG-3^1^), Diva-F (5^1^-ACGGCTGCTGACTGACTACAT-3^1^), Diva-R (5^1^-ACTCTTGGTCATTGGAGAGCA-3^1^), NDPKB-F (5^1^-GAGATCATCAAGCGATTCGAG-3^1^), NDPKB-R (5^1^-CCTGTTTTCACCACATTGAGC-3^1^), β-tubulin-F (5^1^-CAAGATGTCGTCCACCTTCAT-3^1^) and β-tubulin-R (5^1^-CTCAGACACCAGGTCGTTCAT-3^1^).

### Immunofluorescence

The cells were seeded onto sterilized cover slips placed in a 6-well plate. After differentiation, the cells were fixed with 4% paraformaldehyde. Next, the cells were blocked with 5 % horse serum for 1 hour at room temperature and incubated with Diva (sc8740; Santa Cruz), Bcl-B (sc101875; Santa Cruz, USA) and NDPK B (L-14) (sc14789; Santa Cruz, USA), and β-tubulin (sc9104; Santa Cruz, USA) antibodies (All diluted at 1:200) overnight at 4°C. The cells were washed before incubation with secondary antibodies, Alexa Fluor® 488 or Alexa Fluor® 555 (1:200, Invitrogen, USA), for 1 hour at room temperature. The cells were then washed, before they were mounted onto glass slides with Vectashield Mounting Medium with DAPI (Vector Labs, USA). All images were captured using a confocal microscope (Fluoview1000, Olympus, Japan).

### Transfection of PC12 cells

The plasmids from the company Blue Heron Biotechnology (USA) were used. The full length Diva and NDPK B were cloned into pcDNA6A (Invitrogen, USA) with a 6xHis tag. 1x10^6^ cells were seeded into a six well plate and incubated at 37°C and 5% CO_2_ for 24 hours. 4μl of Lipofectamine 2000; 2μg of pcDNA6A, pcDNA6A-Diva and pcDNA6A-NDPK B were diluted in 250μl of OPTI-MEM for 5 minutes respectively. The diluted Lipofectamine 2000 reagent was then added to the diluted plasmids, and the mixture was incubated at room temperature for 25 minutes. The cells were then incubated with the plasmids for 5 hours at 37°C and 5% CO_2._ Thereafter, they were rinsed with 1xPBS once, before fresh media was added.

### Western blot

Equal amounts of protein were separated electrophoretically using SDS-PAGE and then the gel was transferred to 0.45 μm polyvinylidene fluoride (PVDF: Millipore, USA). The membranes were soaked in blocking buffer (5% skimmed milk) for 1 hour at room temperature and then incubated overnight at 4°C with primary antibodies. This was followed by washing with TBS-Tween and incubation with secondary antibodies. For IP studies, Clean-blot reagent (Thermoscientific, USA) was used as it allows clear Western blot detection without IgG bands. This was followed by visualization by the enhanced chemiluminescence (ECL) detection system according to the recommended procedure (Pierce, USA).

### Duolink

The Duolink Kit I (Olink Bioscience, Sweden) was used to study the direct interaction of the proteins. In brief, the wild type, pcDNA6A, pcDNA6A-Diva and pcDNA6A-NDPK B cells were seeded onto 24 well cover slips at 1 x 10^4^ cell density, and treated with NGF for a day. The cells were then fixed in 4% PF for 10 minutes, The cells were then incubated with 1X Duolink blocking stock for 1 hour, before overnight incubation at 4°C with the primary antibodies. Both the anti-goat-plus and anti-rabbit-minus PLA probes were added to the diluent in 1:10 ratio. Next, the cells were washed with 1X TBS-T and the diluted PLA probes were incubated with the samples in a humidity chamber for 2 hours at 37°C. The coverslips were washed and hybridisation solution was added and the samples were incubated for 15 minutes at 37°C. The coverslips were washed with 1X TBS-T and Duolink Ligase was added to the ligation solution at 1:40 dilution, and incubated for 15 minutes at 37°C. The coverslips were washed with 1X TBS-T and Duolink Polymerase was added to the diluted amplification solution at 1:80 dilution, before addition to samples and incubation for 90 minutes at 37°C. The slides were then washed with 1X TBS-T. The detection solution was added to the samples and incubated for 60 minutes at 37°C. The slides were then washed in 2xSSC for 2minutes, 1x SSC for 2 minutes, 0.2x SSC for 2 minutes, 0.02x SSC for 2 minutes and 70% ethanol for 1 minutes before the samples were left to dry. The coverslips were then mounted on slides using Vectashield Mounting Medium with DAPI (Vector Labs, USA) and examined using confocal microscopy (Fluoview1000, Olympus, Japan).

### Brd-U(5-bromo-2^’^-deoxy-uridine) staining

The cells were transfected as mentioned above, before they were reseeded at 50% confluency. The number of proliferating cells was visualized and counted using the BrdU Labeling and Detection Kit I by Roche. In brief, the cell culture medium was aspirated and the cells were incubated with BrdU labeling medium for 30 minutes at 37°C and 5% CO_2._ The cells were washed before they were fixed in ethanol for half an hour at −20°C, washed and then incubated with the Anti-BrdU working solution for 30 minutes at 37°C. After washing, Anti-mouse-Ig-fluorescein working solution was added to the cells and incubated for 30 min at 37°C. After a final 3 washes, the coverslips were mounted onto glass slides with Vectashield Mounting Medium with DAPI (Vector Labs, USA) and photos were taken using the confocal microscope.

### MTS assay

In order to study if the NGF treatment had decreased the proliferation rate of the cells, cell numbers were assayed using CellTiter 96® Aqueous Non-Radioactive Cell Proliferation Assay (Promega), a colorimetric based technique that exploits the ability of viable cells to convert tetrazolium salt into formazan. In brief, cells were plated at a fixed number in 100 μl of cell medium into a 96-well plate and allowed to equilibrate for 2 hours. Thereafter, 100 μl of PMS solution was added to 2 ml of MTS solution, and 20 μl of the PMS/MTS solution was added to each well. The plate was then incubated for 2.5 hours in an incubator at 37°C and 5% CO2. The absorbance for the plate was then read at 490nm in the GENios plate reader (Tecan, Switzerland).

### Flow cytometry

The cells were serum starved so that they would be synchronised. At the designated time point, the cells were washed with 1xPBS, trypsinized and spun down at 1500 rpm, 4°C for 5 minutes. The cell pellet was then resuspended in 70% ethanol and left on ice for a minimum of 2 hours, or incubated at 4°C overnight so as to fix the cells. Before flow cytometry analysis using Beckman Coulter Epics Altra, USA), the ethanol was removed, and the cells washed with 1xPBS once. 1ml of the propidium iodide mixture (1xPBS, 0.1% triton X-100, 0.2mg/ml RNase A, 20ug/ml PI) was then added to the cells, and incubated at 37°C for 15 minutes.

### Statistical analyses

All the experiments were repeated at least in triplicate for statistical purpose, and where appropriate, the data were presented as mean and standard deviation (SD). When comparing only two groups, the quantitative data was statistically evaluated using the Student’s *t*-test. When comparison was made between groups, one-way ANOVA was used. The level of significance was set at p<0.05.

## Abbreviations

Apaf-1: Apoptosis-activating factor-1; Boo: Bcl-2 homologue of ovary; Diva: Death Inducer binding to vBcl-2 and Apaf-1; H1P1R: Hungtington-interacting Protein 1-related; NDPK: Nucleoside Diphosphate Kinase; NHE: Nuclease-hypersensitive element; TCTP: Translationally Controlled Tumor-associated Protein.

## Competing interests

The authors declare that they have no competing interests.

## Authors’ contributions

LQRJ was involved in performing and analysing all experiments. HB conceived of the study. LJ participated in its design and coordination and helped to draft the manuscript. All authors read and approved the final manuscript.
